# Controlling Pandemic Flu: The Value of International Air Travel Restrictions

**DOI:** 10.1371/journal.pone.0000401

**Published:** 2007-05-02

**Authors:** Joshua M. Epstein, D. Michael Goedecke, Feng Yu, Robert J. Morris, Diane K. Wagener, Georgiy V. Bobashev

**Affiliations:** 1 Economic Studies Program, The Brookings Institution, Washington, D. C., United States of America; 2 Statistics and Epidemiology, RTI International, Research Triangle Park, North Carolina, United States of America; 3 Statistics and Epidemiology, RTI International, Rockville, Maryland, United States of America; Medical University of South Carolina, United States of America

## Abstract

**Background:**

Planning for a possible influenza pandemic is an extremely high priority, as social and economic effects of an unmitigated pandemic would be devastating. Mathematical models can be used to explore different scenarios and provide insight into potential costs, benefits, and effectiveness of prevention and control strategies under consideration.

**Methods and Findings:**

A stochastic, equation-based epidemic model is used to study global transmission of pandemic flu, including the effects of travel restrictions and vaccination. Economic costs of intervention are also considered. The distribution of First Passage Times (FPT) to the United States and the numbers of infected persons in metropolitan areas worldwide are studied assuming various times and locations of the initial outbreak. International air travel restrictions alone provide a small delay in FPT to the U.S. When other containment measures are applied at the source in conjunction with travel restrictions, delays could be much longer. If in addition, control measures are instituted worldwide, there is a significant reduction in cases worldwide and specifically in the U.S. However, if travel restrictions are not combined with other measures, local epidemic severity may increase, because restriction-induced delays can push local outbreaks into high epidemic season. The *per annum* cost to the U.S. economy of international and major domestic air passenger travel restrictions is minimal: on the order of 0.8% of Gross National Product.

**Conclusions:**

International air travel restrictions may provide a small but important delay in the spread of a pandemic, especially if other disease control measures are implemented during the afforded time. However, if other measures are not instituted, delays may worsen regional epidemics by pushing the outbreak into high epidemic season. This important interaction between policy and seasonality is only evident with a global-scale model. Since the benefit of travel restrictions can be substantial while their costs are minimal, dismissal of travel restrictions as an aid in dealing with a global pandemic seems premature.

## Introduction

Planning for a possible influenza pandemic is obviously an extremely high priority for the U.S. government. Less obvious, perhaps, is the fact that, in the well-connected world of the 21^st^ century, no country is isolated from the potential spread of infection. Therefore, there is a pressing need to study the global spread of flu to understand the impact of the global epidemic on U.S. preparedness.

Rvachev and Longini [1] developed a deterministic, equation-based SEIR model to study the role of global air travel in the 1968–1969 influenza pandemic. Recently, others have extended that model to update population levels [2], [3], incorporate more recent air travel patterns [2], [3], adjust seasonality parameters [2], [3], add stochasticity to the model [4], and extend it to more cities [4]. In general, these models found that, as compared to 1968, epidemics would spread faster and that the order of cities impacted would change under current air travel patterns. In contrast to Colizza, *et al*. [5], Cooper, *et al*. [4] concluded that international travel restrictions do little to reduce the rate of spread globally. Rather, local interventions aimed at reducing transmission are more likely to reduce the rate of spread. Hollingsworth, *et al*. [6], using a simplified global model, reached similar conclusions.

Here, we argue that international air travel restrictions sometimes could be useful to slow the progression of pandemic flu and sometimes could be harmful. While travel restrictions alone do little to directly ameliorate the pandemic, they can buy time to develop and deliver vaccine and institute a range of powerful nonpharmaceutical interventions (e.g., social distancing, public education, staging of medical equipment), all of which could sharply reduce cases. Of course, travel restrictions can directly decrease the influx of new infected persons into an area. More importantly, the restrictions reduce the probability of an infected individual leaving the area in which an outbreak is developing. Consequently, travel restrictions are one among a range of strategies that could be used to address a global pandemic. In a recent paper, Brownstein, *et al*. [7] have presented supporting evidence, showing that the grounding of airplanes in the United States after September 11, 2001 delayed the dynamics of influenza during the 2001–2002 season by approximately 2 weeks. While air travel restrictions in the United States might have a small impact on domestic disease dynamics due to ground transportation, the global spread, such as a transfer of pandemic flu from Asia or Europe to the United States, could be delayed more significantly by international travel restrictions.

From a public health perspective, it becomes clear that the *main* purpose of travel restrictions is to delay dissemination of the disease until targeted medical and other interventions can be developed and deployed. Even a couple weeks of additional delay is important for the deployment of national and local containment strategies which might not necessarily be related to vaccination *per se*.

To estimate this delay, we model the distribution of first passage times (FPT) for infected persons to the United States. We define the start of the epidemic as the day on which the first 100 individuals are exposed in a single city, and we define the FPT as the number of days from the epidemic start until the first infected individual crosses the United States border. Seemingly small increases in FPT can translate into significant delays in peak incidence times and values. In all pandemic plans, local social interaction restrictions are recommended. Once these are implemented, the course of the epidemic will be altered. In this model, both simultaneous, global restrictions and sequential, city by city restrictions were tested. The impact of the travel restrictions on both the mean FPT to the United States and the full course of the epidemic are considered, as well as the costs of the intervention.

## Methods

### The model

The initial version of our model was based on (and calibrated to) the global influenza model of Rvachev and Longini [1], which was based on an earlier model of Baroyan, Mironov, and Rvachev [8]. We have substantially extended and further developed it by adding seasonality in the disease transmission rate, stochasticity, and several possible disease interventions, such as travel restrictions and vaccination. These interventions can be implemented separately or in combination, and either globally or on a city-by-city basis, to test the effectiveness of different intervention scenarios. In addition, populations are made more detailed by including a nonsusceptible class for those individuals who acquired disease immunity either from exposure during previous epidemics to a similar virus or from vaccination.

The model consists of a set of stochastic difference equations describing the disease dynamics within each city and air travel by individuals from one city to another. Time is measured in discrete units of 1 day. The population of each city is divided into mutually exclusive nonsusceptible (NS), susceptible (S), exposed (E), infectious (I), and recovered (R) classes. We do not estimate deaths, although readers can easily compute them by multiplying our infection levels by any assumed case fatality rate. The exposed period is assumed to coincide with the viral incubation period, and the infectious period is assumed to coincide with the symptomatic period. Infectious persons are assumed not to travel. Within each city, individuals are assumed to be well-mixed. Parameter values are the same for all cities. The parameter values used in the model are given in Table S1. Model equations are given in Text S1.

The model includes 155 major cities around the world, including the cities with the 100 busiest airports, the 100 largest cities worldwide, and the 52 cities in the Rvachev-Longini model. The 155 cities modeled include 34 major U.S. cities. The population and transportation data have been updated to include year 2000–2004 values. Population data are taken from the U.S. Census Bureau, the United Nations Department of Economic and Social Affairs, the Instituto Brasileiro de Geografia e Estatística, and several other sources [9]–[15].

Travel data are taken from OAG statistics on flight schedules provided by L. Amaral [16]. The travel network is made more realistic by allowing asymmetric travel between cities. We have also created a modified travel matrix in which we model travel patterns including more than one leg of travel. The methodology for creating this matrix is described in Text S2.

**Figure 1:**
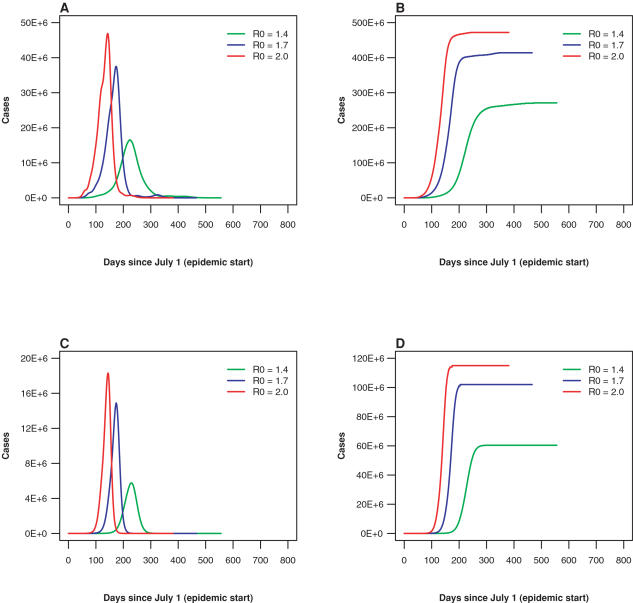
Epidemic severity vs. R_0_ value. The severity and the speed of an epidemic both increase as the value of R_0_ increases. Results are shown for an epidemic starting in Hong Kong on July 1. The actual values of R_0_ are modified by seasonal and geographical factors. (A) Worldwide daily number of infected individuals. (B) Worldwide cumulative number of influenza cases. (C) U.S. daily number of infected individuals. (D) U.S. cumulative number of influenza cases. (green: R_0_ = 1.4, blue: R_0_ = 1.7, red: R_0_ = 2.0)

Natural history parameters for the H5N1 influenza virus align with those used previously [17]–[20]. In particular, the value of R_0_ has been chosen to be 1.7. We have also studied a range of R_0_ values but specifically focused on the values of 2.0 and 1.4, which in combination with 1.7, correspond to the world pandemics of 1918, 1957, and 1968. Seasonality was implemented based on the assumptions that cities within the tropics have peak viral transmission year round, while in cities outside the tropics, transmissibility varies sinusoidally, with peak transmission occurring on January 1 in the northern hemisphere and on July 2 in the southern hemisphere. To avoid abrupt pattern changes at the boundaries of the tropics, we modeled a smooth latitudinal variation of the amplitude by implementing a corresponding sine wave. In Figures 1A–D for the world and U.S. metropolitan areas we show the dependence of the unmitigated epidemic shapes and the totals for the three values of R_0_. Full details are provided in Text S1.

Deterministic models can be effective in describing the mean behavior of a stochastic epidemic, particularly when the number of persons in each disease class is large enough to model the system behavior in terms of population proportions, rather than numbers of individuals. However, in the early stages of an outbreak in a city, when very few exposed or infectious individuals are present, individual actions are important and random factors may easily affect the course of the outbreak. In our model, the realized number of newly infected persons each day within each city is drawn from a Poisson distribution with the mean calculated from the numbers of susceptible and infectious persons in that city and the local, seasonally-adjusted, infectious contact rate. The numbers of individuals in a particular disease state traveling from one city to each of the other cities directly connected to it on a particular day are drawn from a multinomial distribution based on the average daily numbers of travelers from that city and the proportion of the city's population in that disease state on that day.

### Interventions

Travel restrictions are implemented in the model as a reduction in the probability of travel between cities that occurs after a threshold cumulative number of infectious influenza cases has been reached. Sequential restrictions are applied to travel to and from a city that has crossed the threshold of 1,000 cumulative infectious cases. Note that because travel restrictions reduce the travel both into and out of a city, those cities directly connected to a restricted city are also affected by the restrictions, even if they have not yet reached the intervention threshold. We have also considered simultaneous, worldwide interventions, in which travel restrictions are applied to all cities after 1,000 infectious cases have occurred in the initially exposed city. Obviously, one could assume thresholds proportional to city sizes and many other variations.

Vaccination is implemented as a transfer of a percentage of the susceptible population to the permanently nonsusceptible population, and can be implemented as an initial vaccination at time zero (i.e., prevaccination), or as an ongoing, daily vaccination of the population during the epidemic. Daily vaccinations are implemented either simultaneously or sequentially, similar to the imposition of travel restrictions. To more closely parallel travel restrictions, vaccination is implemented at the same time in those cities directly connected to cities which have crossed the intervention threshold. Note, however, that we use the term “vaccination” broadly, to denote simply the product of the number of vaccine courses administered and the effectiveness per course, so that the nonsusceptible population consists of those who have been effectively removed from the susceptible population before becoming infected. Our baseline value for vaccination is that 0.1% of the susceptible population is vaccinated daily.

### Simulation scenarios

We have run a number of scenarios varying the origin of the infection (Hong Kong, London, Sydney), the origination date (January 1, July 1), the level of travel restriction (90%, 95%, 99%), the vaccination strategy (sequential, simultaneous), the initial vaccination level (0%, 10%, 20%), the daily vaccination rate (0.05%, 0.1%, 1%), and the severity of flu transmission (R_0_ = 1.4, 1.7, 2.0). Note that the value of R_0_ that we routinely report in this paper corresponds to a baseline value that is further modified by seasonal variations and latitude. The actual value of R_0_ depends on the location and the season, and thus may be lower than the baseline value. In this paper we illustrate our point by using the most commonly published parameters and scenarios [4], [17]–[22]. In particular, the base set of comparison scenarios uses an epidemic starting in January in Hong Kong with no previously immune individuals, with or without interventions of 95% travel restriction, 0.1% daily vaccination, or a combination of the two. For each scenario, 100 replicates were run to analyze the statistical behavior of the stochastic process. We also present sensitivity to the seasonality by varying the time and location of the origin, leaving the discussion of other scenarios to subsequent manuscripts. We have done a brief sensitivity analysis for the case of a pandemic originating in Hong Kong, in which we assumed that 33% of infectious individuals were asymptomatic, and whose relative infectiousness was 50% of that of symptomatic infectious individuals. These choices are in line with other modeling groups' published assumptions [5], [18], [20]. After adjusting the infectious contact rate in the model to obtain the same effective value of R_0_ = 1.7 as in our main analysis without asymptomatic individuals, we observed no significant qualitative differences in results. We recognize that a more detailed exploration of parameter space may yield more information, and such an analysis may be part of future research.

The model was implemented in AnyLogic™, a Java-based modeling platform developed by XJ Technologies Company Ltd. (www.xjtek.com). Instantaneous model results can be displayed in an animation screen for immediate review and time series results can be written to an external file for further analysis. More details can be found in Text S1 and in the comprehensive model user manual (which is available from the authors upon request). An applet demonstration version of the model can be found on the National Institutes of Health MIDAS (Models of Infectious Disease Agent Study) portal, at www.midasmodels.org. For particular runs, this model offers a number of visualizations: the global spread displayed on a world map (see Figure S1), city-specific levels of infection, and a global time series of epidemic waves.

## Results

**Figure 2:**
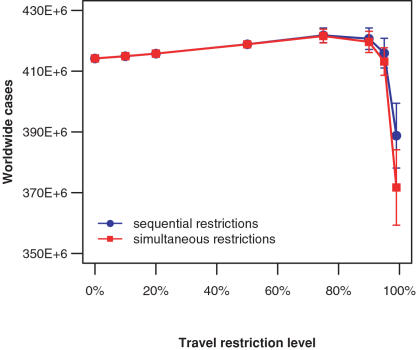
Effects of travel restrictions on epidemic severity. High levels of international travel restrictions are necessary to reduce the total number of infected individuals worldwide. There is little difference in effect between sequential, city-by-city implementation of travel restrictions and simultaneous, worldwide implementation. Results are shown for an epidemic with R_0_ = 1.7 starting in Hong Kong on July 1. (blue: sequential travel restrictions; red: simultaneous travel restrictions, mean values shown, error bars = 95% confidence intervals)

Consistent with previous work [4], [6], [17], [18], our study shows that international travel restrictions *per se* do not provide an effective way to contain the epidemic. In Figure 2 we show that to significantly reduce the total number of cases worldwide, it is necessary to implement drastic restriction measures, by reducing the flight volume by at least 95%. As expected, we did not see a significant difference between sequential and simultaneous travel restriction (differences in the total numbers of cases were less than 5%). Before the virus reaches a given region, travel restrictions within that region have no effect on the spread of the epidemic, and thus would be unnecessary. Once cities in a region have passed the epidemic threshold for implementing sequential travel restrictions, implementation is the same in that region whether the restrictions are sequential or simultaneous. Thus, travel conditions for infected persons in given cities would be different in the two scenarios only during the time between the initial arrival of the virus and the crossing of the travel restriction implementation threshold. Therefore we will focus on the more realistic and feasible sequential travel restriction policy. This approach is also far less disruptive economically than simultaneous closures. Thus, here is a policy choice where two approaches (simultaneous and sequential) are indistinguishable epidemiologically but are quite distinct economically, and we chose the less disruptive. This type of result argues for more explicit inclusion of economic considerations in the comparison of mitigation strategies.

Table 1 shows that 95% travel restrictions can delay the initial spread of the epidemic, as measured by the number of cases after 6 months. The difference between the cumulative numbers of cases reflects the fact that, because of the high growth rate of the epidemic, a delay of even few weeks can cause a large difference in the cumulative number of cases at 6 or 12 months. The values of the total numbers of cases at the end of the epidemic are less dependent on the effect of travel restriction and may, in fact, increase. The total number of epidemic cases is more dependent on the interaction of the delay (due to travel restrictions) and seasonality, as discussed further below. Note that because we used only the largest metropolitan areas, the results presented in figures and tables of this paper reflect the population only in those metropolitan areas, and not in the entire United States or the world.

**Table 1:** Worldwide metropolitan cases, with and without 95% travel restrictions implemented sequentially after the first 1,000 cases have been identified in each city, for an epidemic with R_0_ = 1.7.

Location and Time of InitialCases	Travel Restrictions Implemented	Total Metropolitan Cases Worldwide after 6 Months		Total Metropolitan Cases Worldwide after 12 Months		Total Metropolitan Cases Worldwide at End of Epidemic^1^	
		mean	sd	mean	sd	mean	sd
Hong Kong - Jan 1	no	193,609,206	4,345,032	293,636,107	3,096,894	358,390,361	1,342,560
	yes	81,531,156	9,783,597	331,162,274	3,836,716	391,746,313	2,736,224
Hong Kong - July 1	no	323,819,238	4,071,117	414,093,710	255,211	414,198,937	244,499
	yes	132,230,576	9,451,456	409,718,662	1,974,674	415,947,262	2,462,781
London - Jan 1	no	216,643,706	2,791,062	275,413,403	2,270,138	347,348,752	2,986,580
	yes	118,523,844	10,690,524	321,370,868	5,570,406	385,633,413	3,058,182
London - July 1^2^	no	22,673,116	57,638,959	81,867,867	164,641,526	82,021,371	164,941,514
	yes	7,134,433	19,098,146	61,749,309	141,663,297	67,074,165	149,098,629
Sydney - Jan 1	no	80,356,144	25,615,355	335,303,211	10,261,001	373,149,982	2,987,185
	yes	33,068,217	18,255,000	327,274,492	10,724,921	406,597,417	5,940,327
Sydney - July 1	no	298,429,077	6,434,137	417,607,112	400,989	417,718,338	416,499
	yes	94,823,730	13,494,412	406,339,496	2,846,810	412,396,914	3,138,013

1 The end of the epidemic is determined when there are no further cases worldwide.

2 These data represent means and standard deviations for all 100 runs, including the runs in which the disease did not develop a pandemic state and did not reach the U.S.

Note: The data are presented for only the 155 major cities, not the entire world population.

In Figures 1A–D for the world and U.S. metropolitan areas, we show several unmitigated epidemic shapes and totals for three values of R_0_. In Table 2, the mean FPT is given for a number of scenarios, for an epidemic with R_0_ = 1.7. When travel restrictions are imposed, the FPT increases by two to three weeks when the outbreak originates in Hong Kong (from 18 days to 31 days) or Sydney (from 7 days to 27 days). There is no delay in FPT when the outbreak originates in London. These delays are larger for smaller values of R_0_; for example, for an R_0_ = 1.4 the delay in FPT from Hong Kong to the U.S. due to travel restrictions increases to 20–23 days (data not shown).

**Table 2:** Mean First Passage Times (in days) to the metropolitan U.S. under travel restriction and vaccination intervention scenarios.

Location and Time of Initial Cases	No Intervention		95% Travel Restriction Only		0.1% Daily Vaccination Only		Both Travel and Vaccination	
	mean	sd	mean	sd	mean	sd	mean	sd
Hong Kong - Jan 1	17.58	7.23	31.12	12.44	17.75	5.02	30.40	13.17
Hong Kong - July 1	17.86	6.17	31.33	14.42	18.94	7.07	30.06	14.19
London - Jan 1	5.50	3.94	5.50	3.88	5.34	4.47	5.91	4.44
London - July 1^1^	16.26	32.97	16.15	33.20	23.86	40.35	25.95	44.95
Sydney - Jan 1	34.91	17.80	62.03	33.50	32.96	15.85	69.07	33.79
Sydney - July 1	14.63	6.23	21.32	14.60	14.20	6.16	23.10	14.09

**Base R_0_ = 1.7.**

1 These data represent means and standard deviations for all 100 runs, including the runs in which the disease did not develop a pandemic state and did not reach the U.S.

Vaccination alone, even at low rates, reduces the total number of cases worldwide and in the United States (data not shown). As expected, vaccination reduces the effective R_0_, which leads to the reduction of the total number of cases, and also increases the duration of the epidemics. The FPT, however, is little affected by the vaccination-only intervention (see Table 2), primarily because of the implementation schedule. Vaccination strategies might not be very effective in the early stages of the epidemic because of poor vaccine matching, lack of delivery methods, low public awareness, etc.

**Figure 3:**
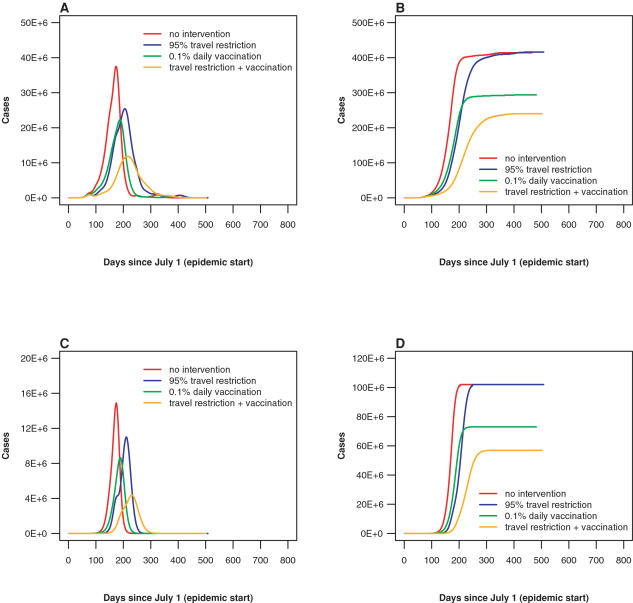
Epidemic severity vs. intervention policy. The speed and severity of an epidemic can be reduced by implementation of travel restriction and vaccination policies. Implementing both travel restrictions and vaccination can have a greater effect than implementing either policy alone. Results are shown for an epidemic with R_0_ = 1.7 starting in Hong Kong on July 1. (A) Worldwide daily number of infected individuals. (B) Worldwide cumulative number of influenza cases. (C) U.S. daily number of infected individuals. (D) U.S. cumulative number of influenza cases. (red: no intervention, blue: sequential 95% restriction of international travel, green: daily vaccination of 0.1% of susceptible population, orange: both travel restriction and vaccination)

Table 3 and Figures 3A–D illustrate the interaction of travel delay and vaccination strategies in the United States. Parallel to Table 1, in Table 3 we present the numbers of U.S. metropolitan cases at 6 months, at 12 months, and at the end of the epidemic. Both vaccination and travel restrictions have a strong effect on the reduction in the number of cases at 6 and 12 months. The reduction varies from 3 to 5 fold at 6 months. While vaccination truly reduces the total numbers of cases, travel restrictions provide the additional delay in epidemic growth and the potential to vaccinate more individuals. The overall value of that delay in combination with vaccination can be seen in the reduction of the total number of epidemic cases. For example, for the case of an epidemic starting in July in Hong Kong, the total number of metropolitan cases in the United States is 102.4 million; 0.1% daily vaccination reduces this number to 73.0 million, and adding sequential travel restriction to the vaccination policy reduces it further, to 56.9 million.

**Table 3:** Mean cumulative number of cases in the metropolitan U.S. under travel restriction and vaccination intervention scenarios.

Location and Time of Initial Cases	Intervention^1^	Total Metropolitan U.S. Cases 6 Months after the Start of the Epidemic		Total Metropolitan U.S. Cases 12 Months after the Start of the Epidemic		Total Metropolitan U.S. Cases at the End of the Epidemic^2^	
		mean	sd	mean	sd	mean	sd
Hong Kong - Jan 1	N	18,245,753	1,657,562	62,118,714	2,517,623	82,833,403	318,722
	TO	2,951,395	1,765,465	90,173,754	1,667,679	96,429,042	1,107,950
	VO	6,017,992	820,342	13,231,241	238,007	16,386,410	494,161
	TV	812,576	700,550	8,006,939	1,861,928	17,910,022	2,491,967
Hong Kong - July 1	N	83,701,712	1,004,370	102,368,352	76,848	102,368,456	76,846
	TO	18,913,221	1,474,799	102,418,028	409,462	102,418,055	409,465
	VO	32,642,187	992,303	72,958,924	216,288	73,008,133	247,663
	TV	3,942,933	837,907	56,928,367	2,087,690	56,928,594	2,087,572
London - Jan 1	N	30,099,814	1,256,785	41,865,074	1,378,565	76,508,738	1,527,186
	TO	13,591,127	2,772,253	77,390,536	4,173,168	92,464,670	994,422
	VO	12,660,235	987,928	14,420,437	737,109	14,806,721	621,159
	TV	4,344,538	1,311,592	8,600,742	968,822	12,602,370	524,534
London - July 1^3^	N	5,277,589	16,653,990	20,382,433	40,984,845	20,382,469	40,984,918
	TO	1,030,904	5,350,070	15,484,489	35,542,037	16,277,110	36,239,497
	VO	1,482,819	5,799,144	12,336,496	27,433,567	12,336,532	27,433,647
	TV	757,059	2,828,242	10,225,818	22,137,451	10,231,246	22,140,760
Sydney - Jan 1	N	4,032,772	4,944,879	79,002,872	4,418,787	84,376,383	731,748
	TO	1,646,404	2,792,800	82,209,426	6,160,091	101,034,551	3,632,634
	VO	1,163,532	1,546,781	21,035,146	3,261,573	33,057,868	5,607,727
	TV	248,120	622,647	6,055,356	2,372,328	26,395,864	7,881,035
Sydney - July 1	N	82,454,611	2,075,752	102,519,057	105,857	102,519,138	105,861
	TO	17,977,729	1,887,605	101,981,636	556,065	101,981,640	556,065
	VO	34,503,408	1,997,174	74,304,850	315,807	74,305,013	315,807
	TV	4,954,351	1,428,691	58,365,274	2,605,232	58,365,422	2,605,185

**Base R_0_ = 1.7.**

1 N: no intervention, TO: 95% travel restriction only, VO: 0.1% vaccination only, TV: both 95% travel restriction and 0.1% vaccination

2 The end of the epidemic is determined when there are no further cases worldwide.

3 These data represent means and standard deviations for all 100 runs, including the runs in which the disease did not develop a pandemic state and did not reach the U.S.

### Travel restrictions and seasonality

**Figure 4:**
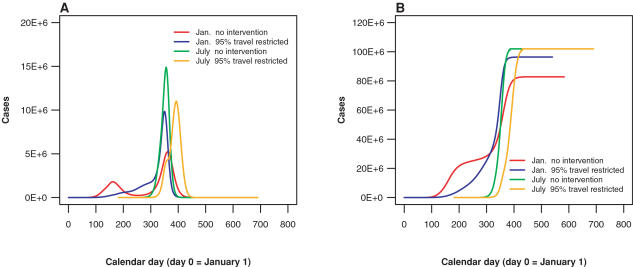
Interaction between disease seasonality and travel restriction. The timing of an outbreak can greatly influence the effects of international travel restrictions on the severity of the epidemic in a region such as the United States. Results are shown for epidemics with R_0_ = 1.7 beginning in Hong Kong on either January 1 or July 1. For an epidemic beginning in January, the initial epidemic wave in the United States is suppressed, although without other interventions, the second epidemic wave would be more severe. It is thus important to implement additional measures during the time gained. For an epidemic beginning in July, the delay in the epidemic is much smaller, but the overall severity is reduced. (A) U.S. daily number of infected individuals. (B) U.S. cumulative number of influenza cases. (red: January 1 epidemic start in Hong Kong with no intervention, blue: January 1 start in Hong Kong with sequential 95% restriction of international travel, green: July 1 epidemic start in Hong Kong with no intervention, orange: July 1 start in Hong Kong with sequential 95% restriction of international travel)

An important result of the model is that the delay of viral introduction caused by travel restrictions may interact with seasonality to cause a larger initial epidemic peak or total number of infected individuals in a region such as the United States. This can happen when the restrictions push the local epidemic outbreak into a period of higher seasonal transmission of the virus, causing it to spread more rapidly through the local population. By the same token, depending on the timing of the initial outbreak in the world, delays caused by travel restrictions can shift introduction of the virus to a period of lower transmissibility, making the local outbreak less severe. Thus travel restrictions alone may have either a positive or a negative local effect. Tables 1 and 3 reflect such interactions and Figures 4A and 4B provide visual illustration. In the United States, the total cumulative number of cases for an epidemic with 95% travel restrictions imposed is approximately equal to that of the unmitigated epidemic, when the epidemic begins in July, regardless of its initial location in the world. However, if the epidemic is initiated in January, the delayed FPT results in a slow disease introduction into the United States. As a result, the spring epidemic is minor and is followed by a large epidemic in the fall, during the beginning of the high contact rate season. The resulting epidemic has substantially more cases, with an increase of about 12%, 16%, or 21%, depending on whether the initial source of infection is Sydney, Hong Kong, or London. As shown in Figure 4A, the epidemic in the United States has 2 peaks: the first, small peak in summer and the second, large peak in winter. With travel restrictions imposed, the first peak is smaller and thus easier to contain than the first peak in an epidemic without travel restrictions. However, if the small peak in the travel restriction scenario is not managed, the following winter peak is higher and the total number of infected persons is larger than in the unrestricted scenario. This result emphasizes the need to implement other disease containment and reduction policies during the achieved delay. It also highlights the utility of global models in capturing the interaction of policies (such as travel restrictions) and planetary scale dynamics (such as seasonality).

### Disease transmission rates

**Figure 5:**
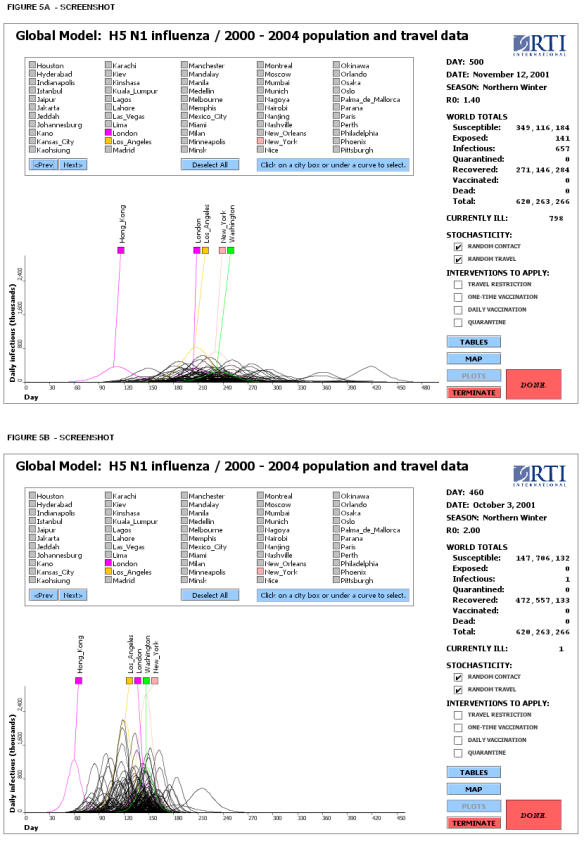
Potential synchronization of local epidemics depending on the rate of disease transmission. Screenshots showing that with higher values of R_0_, individual cities' epidemic peaks are more clustered in time and the number of infected persons is much higher. (A) Time series diagram for major metropolitan areas for an uncontrolled influenza epidemic with R_0_ = 1.4. (B) Time series diagram for an epidemic with R_0_ = 2.0.

A number of factors can modify the results of the simulations. For example, with a higher value of R_0_, a global epidemic would be more “synchronized” (individual cities' peaks would be more clustered) and the delay would be less pronounced. This might be the case in the early stages of an epidemic, when the public is not yet aware of basic contact reduction measures that reduce the effective reproduction number of the virus. However, with the implementation of such measures, the disease transmission rate could be reduced and the delays would become more pronounced. In Figures 5A and 5B we illustrate the temporal spread of the global epidemic for the metropolitan areas under different values of R_0_. For example, if the value of R_0_ is lowered from 1.7 to 1.4 by either self-isolation or other means of reducing contact rates, the delay due to travel restrictions will be increased to 20–23 days, giving public health officials more time to prepare for the upcoming epidemic. The value of R_0_ can be crudely calculated from the early stage of the epidemic growth curve from an equation such as
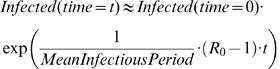
, [23].

If R_0_ is so estimated during the early stages of the epidemic, then it would be possible to predict the amount of time available to increase public health preparedness before the epidemic strikes the United States.

### Cost to the U.S. of air travel restrictions

In deciding whether to adopt a policy such as the imposition of international air travel restrictions, one must compare benefits to costs. The foregoing analysis suggests very strongly that restrictions on international air passenger travel can be of substantial benefit to the U.S. It is not widely appreciated that the associated cost is minimal. To economists, a *cost* is the Gross National Product (GNP) loss of the control measure: the value of all economic activity foregone because of its imposition. A central problem in estimating these costs is that for many activities *substitutions* are possible. So, if one were to close business air travel, the same economic activity (e.g., trades, contracts) may take place electronically, with no loss to total output. Likewise, people who had planned to fly to leisure destinations may decide to drive or take a train, or to substitute a new destination, again with no welfare loss. To estimate the full cost in an orthodox fashion, one could develop the full computable general equilibrium model of the entire economy with specific sectors (notably transportation) explicitly represented. One would run that model to equilibrium with all airlines operative. One then would shut down the airlines and rerun the model to (a presumably different) equilibrium. Then one would compare the equilibria and assess the difference. For our present purposes, this is neither feasible nor necessary. Rather, we will develop a conservative upper bound on the cost of the proposed intervention, and show that it is very modest in GNP terms.

First, of the 155 international cities included in the analysis, 34 are U.S. cities with major airports. These account for the activity of airlines classified in the Bureau of Transportation Statistics as Major carriers. Ranked below them in size are the National, Large Regional, Medium Regional, Small Certified, and Commuter carriers [24]. The Major carriers account for just over 85% of the industry's activity [24]. However, for our bounding calculation we will assume that when they shut down, the entire system shuts down for passenger travel, implicitly eliminating a variety of alternative means of air travel (e.g., using multiple legs on smaller carriers). This conservative assumption overstates the GNP loss. We do assume that freight and cargo air continue to operate, presumably with anti-viral prophylaxis and continuous screening of pilots and crews. Even were air cargo to cease, there would be shipping and land transportation as substitutes, so GNP loss would again be minor. As for private flights for non-business purposes, people may drive or take rail to leisure destinations, or may change destinations. The true cost of an imposed closure is further complicated by the fact that many people would endogenously stop flying, as occurred during the SARS outbreak in 2003, so the loss *beyond* this endogenous response is difficult to estimate with precision.

With all of this information as background, we estimate the cost of closing the Major airlines as if that were the equivalent of shutting down the entire system, and we will cost it as a simultaneous shutdown, although as discussed above, we model a sequential shutdown. Under these worst case assumptions, the estimated cost falls between $93 billion and $100 billion *per annum*, extrapolating from the complete and immediate cessation that occurred after 9/11 [25]. Thus, even under the worst case assumption that this economic activity is simply sacrificed, the cost is still around 0.8% of the $12 trillion U.S. GNP per year [26].

Labor deserves a separate discussion. First, the impact on labor depends on whether the economy is operating at full employment. If not (as in the U.S. at present), many workers (managers, executives, baggage handlers, agents, mechanics, etc.) would find alternative employment (i.e., there would be some factor mobility). For conservatism's sake, let us assume no such mobility. Roughly 60% of the airline industry remains non-unionized. These individuals received no severance pay after the layoffs of 9/11 and would likely be treated similarly in a pandemic flu shutdown. Severance packages are unlikely; hence, labor costs will not likely weigh heavily on the calculation of costs. We are not condoning this treatment of workers, merely reporting the likely GNP impact. Indeed, a more generous labor policy is altogether feasible. The Senate Joint Economic Committee estimates that “a government funded severance package that covered 100 percent of wages and benefits would cost roughly $500 million per month.” [25] That is $6 billion per year. If this amount were added to the price tag of our policy, the total would rise from $100 billion to $106 billion, increasing the entire cost from 0.8% of GNP to perhaps 0.9%, still very far from ruinous.

In summary, considering substitution possibilities, and even including labor compensation, it is extremely difficult to drive the cost of air travel restrictions beyond 1% of the U.S. GNP *per annum*. (Rough private calculations communicated to the authors by transportation economists using various estimation methods are approximately half this magnitude, reinforcing our claim that this is a plausible upper bound.)

## Discussion

We have presented a study of the impact of a number of interventions on the mean first passage time of a pandemic virus to the United States and on the total number of cases both worldwide and in the U.S. We have shown that although international travel restrictions alone will not contain a pandemic, they can buy time in which to take important steps. Our results suggest that the delay can be significant (about 2 to 3 weeks). Although this is not enough time to develop and produce large quantities of a vaccine, from a public health perspective, a delay of even 1 or 2 weeks can be a big help in preparing for vaccination, developing public awareness, instituting social distancing, organizing vaccination centers, and preparing other means of disease containment. One should also note that the effect of the travel restrictions is not limited to delaying the initial disease introduction. Restrictions also help to limit continuous reintroduction of the disease to the United States, and thus allow development of more efficient local containment measures.

The impact of travel restrictions on the total number of cases in an epidemic is roughly comparable to vaccination of a substantial portion of the population. However, because of the delay in FPT, the number of cases at intermediate time points such as 6 or 12 months following the initial outbreak of the epidemic worldwide can be substantially reduced when travel restrictions are used.

A number of factors could modify the effects of the delay caused by travel restrictions. Seasonality is one of them. Seemingly counterintuitively, due to the interaction with the global seasonality of influenza, travel restrictions alone may lead to a higher number of total cases in a given region than would an unmitigated epidemic. This occurs because the increased FPT may delay the regional introduction of the virus until the influenza season. For example, an outbreak in Hong Kong occurring in January would lead to a slow epidemic start in the United States in the spring, when the seasonal transmission rate is low. As the seasonal transmission rate increases around September, one would expect to see a large epidemic outbreak. Any delay of the epidemic introduction in the United States would only push the disease into a season with a higher effective reproduction number. Conversely, when an epidemic starts in Hong Kong in July and becomes visible in the United States around October it peaks around February. Any delay in introduction will push the epidemic out of the high transmissibility season and thus reduce the total number of cases. The actual seasonality pattern might vary slightly depending on how seasonality is introduced; however, the general relationship between seasonality and the delay will hold and is worth considering when planning prevention measures.

Because of seasonality and travel patterns, first passage times differ greatly depending on the location of the outbreak. For instance, for January outbreaks, the mean FPT to the U.S. is 18 days from Hong Kong, 6 days from London, and 35 days from Sydney. January is outside influenza season in Sydney, so the outbreak requires more time to reach a level such that a number of travelers would carry the infection to other cities. The short FPT to the United States from London reflects the heavy volume of air travel between London and the United States.

In the study we used a conservative operational estimate for the imposition of intervention policies of 1,000 total infectious cases. One could use 500 total cases or even just a single case; however, the use of a single case as a signal of an epidemic start might be dramatically misleading because of the stochasticity at the small size of the infectious population. As a modeling assumption, the choice of a single fixed threshold was based on parsimony. It seems likely that different countries would have different thresholds. However, lacking detailed data to support city-specific estimates, we chose not to add further model complexity based on undocumented hypotheses. The base case value of 1000 infectious cases as the threshold for implementation of travel restrictions was meant as a conservative bound; that is, we chose a relatively high number to ensure that the analysis was not biased in favor of travel restrictions. Obviously, they look better the earlier they are implemented. This threshold should not be confused with the onset of local containment measures. Presumably these could begin earlier, and after the first cases are reported anywhere, vigilance will likely increase everywhere. That is, the country response thresholds should fall as the disease spreads. This makes our use of the constant threshold more conservative.

Our simulations show (see Figures 4A and 4B) that the delay between the initial cases and the epidemic peak in the United States is on the order of 150 days. However, at the beginning of the epidemic the growth rate is almost exponential and the time difference between 500 and 1000 cases is on the order of a few days. This result emphasizes the importance of early surveillance and the need to have a clear plan for public health officials to implement in the first two or three weeks after initial detection, before the epidemic reaches the United States. This is especially important if an epidemic reaches the United States in spring or summer, because if the disease is not eliminated until the high season it could be disastrous. This is a significant period to consider for public health planning.

In the simulations presented here, we have used a single-leg travel matrix, primarily to be consistent with other authors [1]–[4]. However, we have also calculated a more realistic travel matrix which accounts for the fact that only 60% of travelers travel one leg to reach their final destination, 37% travel two legs, and only 3% travel three legs or more. Others have also developed a two-leg travel matrix [5], [21], [22]. Although travel patterns change when using the multileg travel matrix, the main transmission paths remain the same as for the single-leg travel matrix. For example, the most likely travel path from Hong Kong to the United States is a direct flight to Los Angeles. The results for the two-leg travel matrix remain qualitatively similar, with some modification of the mean and total values. The mean FPT to the U.S. for a January Hong Kong epidemic without travel restrictions is the same (18 days), while with 95% travel restrictions the mean FPT is 2 days less (29 days vs. 31 days). This result is expected, because the travel patterns have strong interactions with the travel restrictions. For example, with multileg travel, the number of connections for each city increases, because the matrix also includes cities reachable in two steps. The longer the travel path, the closer sequential travel restrictions become to simultaneous travel restrictions. Since we have not observed a large difference in effect between simultaneous and sequential restrictions, we should not expect a large qualitative difference between using the single-leg and the multileg travel matrices. Furthermore, many major metropolitan areas are directly connected and therefore can be reached with only one leg of travel. This fact justifies the selection of the relatively small number of airports (155 out of 3,100) which cover most of the connections between the regions.

### Limitations

This mathematical model is focused on the description of the disease spread across the continents and has a number of limitations. The model is based on the largest metropolitan areas. It does not include the heterogeneous populations around these cities and in rural areas. Other types of heterogeneity, such as population age structure or social networks and the consequent differences in transmission probability are not considered. Further, the model does not include ground transportation.

Our mathematical model does allow one to evaluate the impact of travel restrictions combined with other types of interventions, such as quarantine, self-isolation, wearing masks, closing schools, etc. We have presented a number of scenarios illustrative of the interactions between location, seasonal timing, travel restrictions, and vaccination. Our future work is focused on more complex scenarios involving other disease characteristics and other factors effectively reducing disease transmission beyond vaccination-type strategies.

### Cost-benefit

Economically, a 1-year total ban on international and major U.S. domestic air passenger travel is estimated to cost the United States less than 1% of GNP. Because our model predicts that regionally implemented sequential travel restrictions may be just as effective as simultaneous global restrictions, we expect the direct economic impact would be even smaller. Given that the benefits of air travel restrictions can clearly be substantial, while the costs are clearly minimal, their dismissal is premature; the approach deserves serious consideration as an adjunct to other direct disease control measures.

## Supporting Information

Text S1 Model Equations and Initial Conditions
(0.31 MB DOC)

Table S1 Parameters and Values for the Model that Do Not Vary over Time
(0.09 MB DOC)

Text S2 Modifications to the Travel Matrix to Account for Multiple Legs of Travel
(0.16 MB DOC)

Figure S1 A user can select one of three visualization screens: a world map view, time series plots, or numeric tables for each of the cities. Before running the model, one can choose to produce stochastic or deterministic runs and choose the types of intervention. Each spot on the map corresponds to a metropolitan area. Clicking on a spot will display the city name and a snapshot of the city disease status. Arrows link each infected city with its initial source of infection.
(0.45 MB TIF)

## References

[1] Rvachev LA, Longini IM (1985). A mathematical model for the global spread of influenza.. Math Biosci.

[2] Grais RF, Ellis JH, Glass GE (2003). Assessing the impact of airline travel on the geographic spread of pandemic influenza.. Eur J Epidemiol.

[3] Grais RF, Ellis JH, Kress A, Glass GE (2004). Modeling the spread of annual influenza epidemics in the U.S.: The potential role of air travel.. Health Care Manag Sci.

[4] Cooper BS, Pitman RJ, Edmunds WJ, Gay NJ (2006). Delaying the international spread of pandemic influenza.. PLoS Med.

[5] Colizza V, Barrat A, Barthelemy M, Valleron AJ, Vespignani A (2007). Modeling the worldwide spread of pandemic influenza: Baseline case and containment interventions.. PLoS Med.

[6] Hollingsworth TD, Ferguson NM, Anderson RM (2006). Will travel restrictions control the international spread of pandemic influenza?. Nature Med.

[7] Brownstein JS, Wolfe CJ, Mandl KD (2006). Empirical evidence for the effect of airline travel on inter-regional influenza spread in the United States.. PLoS Med.

[8] Baroyan OV, Mironov GA, Rvachev LA (1981). An algorithm modeling global epidemics of mutant origin.. Programming and Comput Software.

[9] Brinkhoff T (2005). *Mato Grosso City Population*.. http://www.citypopulation.de/Brazil-MatoGrosso.html.

[10] ESRI (2005). ArcGIS 9 World, Europe, Canada, and Mexico: 1996, 1998, Winter 1993/1994. [Computer software and data files 20000101, 2000, 20000225, 20010128, 20000612, 20020314, 20021115, 2000, 2003].

[11] Helders S (2005). *World Gazetter*.. http://www.world-gazetteer.com.

[12] Instituto Brasileiro de Geografia e Estatística (IBGE) (2006). http://www.ibge.gov.br.

[13] Mongabay. com (2004). http://population.mongabay.com.

[14] Population Division of the Department of Economic and Social Affairs of the United Nations Secretariat, World Population Prospects (2004). *World Urbanization Prospects: The 2003 Revision Population Database*.. http://esa.un.org/unup.

[15] Population Division, U.S. Census Bureau (2004). *Table 1. Annual Estimates of the Population of Metropolitan and Micropolitan Statistical Areas: April 1, 2000 to July 1, 2004* (CBSA-EST2004-01).. http://www.census.gov/population/www/estimates/Estimates%20pages_final.html.

[16] Guimerà R, Mossa S, Turtschi A, Amaral LAN (2005). The worldwide air transportation network: Anomalous centrality, community structure, and cities' global roles.. Proc Natl Acad Sci U S A.

[17] Ferguson NM, Cummings DAT, Cauchemez S, Fraser C, Riley S (2005). Strategies for containing an emerging influenza pandemic in Southeast Asia.. Nature.

[18] Longini IM, Nizam A, Xu S, Ungchusak K, Hanshaoworakul W (2005). Containing pandemic influenza at the source.. Science.

[19] Ferguson NM, Cummings DAT, Fraser C, Cajka JC, Cooley PC (2006). Strategies for mitigating an influenza pandemic.. Nature.

[20] Germann TC, Kadau K, Longini IM, Macken CA (2006). Mitigation strategies for pandemic influenza in the United States.. Proc Natl Acad Sci U S A.

[21] Colizza V, Barrat A, Barthélemy M, Vespignani A (2006a). The role of the airline transportation network in the prediction and predictability of global epidemics.. Proc Natl Acad Sci U S A.

[22] Colizza V, Barrat A, Barthélemy M, Vespignani A (2006b). The modeling of global epidemics: Stochastic dynamics and predictability.. Bull Math Biol.

[23] Anderson RM, May RM (1991). *Infectious Diseases of Humans: Dynamics and Control*..

[24] Bureau of Transportation Statistics, *Air Carrier Financial Statistics*, U.S. Government. July 11, 2002.

[25] United States Senate, Joint Economic Committee, *Assessing Losses for the Airline Industry and its Workers in the Aftermath of the Terrorist Attacks*. October 31, 2001.

[26] U.S. Department of Commerce, *Survey of Current Business*. Vol 85, No. 19. October 2005.

