# Supplementary material for: Controlling Pandemic Flu: The Value of International Air Travel Restrictions
Source: PLoS One. 2007 May 2;2(5):e401. doi: 10.1371/journal.pone.0000401 (PMC1855004; doi:10.1371/journal.pone.0000401)
Supplement: Text S1 — Model Equations and Initial Conditions (0.31 MB DOC) [file pone.0000401.s001.doc]

**Text S1 – Model Equations and Initial Conditions**

Let $NS_{i}(t)$, $S_{i}(t)$, $R_{i}(t)$, and $D_{i}(t)$ be the number of individuals in city $i$ on day $t$ who are in the Nonsusceptible, Susceptible, Recovered, and Dead states respectively. Similarly, let $E_{i}(\tau,t)$and $I_{i}(\tau,t)$be the number of individuals in city $i$ who are in the Exposed and Infectious states on day $t$ and who were infected $\tau$ days earlier, on day $t-\tau$. Then the total population, $T_{i}(t)$, of city $i$ on day $t$ can be expressed as:

$T_{i}(t)=NS_{i}(t)+S_{i}(t)+\sum_{\tau=0}^{\tau_{1}} E_{i}(\tau,t)+\sum_{\tau=0}^{\tau_{2}} I_{i}(\tau,t)+R_{i}(t)+D_{i}(t)$ (1)

Following Rvachev and Longini (1985), we also define $W_{i}(t)$to be the number of individuals who become Infectious in city $i$ on day $t$, that is, the daily morbidity, and define $B_{i}(t)$ to be the number of newly Infectious individuals in city $i$ on day $t$ who are reported to the health authorities.

### *Transition Probabilities of Disease*

The set of all individuals who were initially infected on day $t-\tau$ can be partitioned into those who are in the Exposed, Infectious, or Removed (Recovered or Dead) states $\tau$ days later, on day $t$. Let $L_{1}$ be the length of time that a given person is in the Exposed state, and let $L_{2}$ be the total length of time that that person is in either the Exposed or the Infectious state. Let $\gamma(\tau)$ be the probability that an individual becomes Infectious on day $\tau+1$, given that that person was still in the Exposed state on day $\tau$. Let $\delta(\tau)$ be the probability that a person is Recovered or Dead on day $\tau+1$, given that that individual was still in the Infectious state on day $\tau$. Then, following Rvachev and Longini,

$\gamma(\tau)=P\left(L_{1}\leq\tau+1|L_{1}>\tau\right)=\frac{f(\tau)-f(\tau+1)}{f(\tau)} ,\text{ for }f(\tau)>0,\text{ }\tau=0, 1, \ldots, \tau_{1},$ (2)

$\delta(\tau)=P\left(L_{2}\leq\tau+1|L_{1}\leq\tau<L_{2}\right)=\frac{h(\tau+1)-h(\tau)}{g(\tau)},\text{ for }g(\tau)>0,\text{ }\tau=0, 1, \ldots, \tau_{2},$ (3)

where $f(\tau)$, $g(\tau)$, and $h(\tau)$ are the probability distributions for being in the Exposed, Infectious, and Removed states, respectively:

$f(\tau)=P\left(L_{1}>\tau\right),\text{ for }\tau=\text{0,} \text{1,} \ldots\text{,} \tau_{1},$ (4)

$g(\tau)=P\left(L_{1}\leq\tau<L_{2}\right),\text{ for }\tau=\text{0,} \text{1,} \ldots\text{,} \tau_{2},$ (5)

$h(\tau)=P\left(L_{2}\leq\tau\right),\text{ for }\tau=\text{0,} \text{1,} \ldots\text{,} \tau_{2}+1 ,$ (6)

with

$f(\tau)+g(\tau)+h(\tau)=1,$ $f(0)=1,$ and $g(0)=h(0)=0$ . (7)

These transition probabilities are used to estimate evolution from one disease state to another. Equation (7) follows from the assumption that $f(\tau)$, $g(\tau)$, and $h(\tau)$ partition those individuals who have been infected, and from the assumption that an individual’s Exposed period lasts at least 1 day.

Mortality due to the disease is modeled as a fraction $d$of the individuals who are removed from the Infectious state. Thus, for a person in state $I_{i}(\tau,t)$, the probability of dying is $d\cdot\delta(\tau)$, while the probability of recovery is $(1-d)\cdot\delta(\tau)$.

### *Infection of Susceptibles*

Under the assumption of homogeneous mixing within a city, contacts between Susceptible and Infectious individuals are uniformly distributed. Let $\lambda$ be the daily infectious contact rate, defined as the average number of individuals with whom an Infectious individual will make sufficient contact to pass infection in a day. Then the average number of new infections caused by one Infectious person in city $i$ on day $t$ is proportional to the number of Susceptible individuals in that city on that day, and is equal to $\lambda\cdot S_{i}(t)/T_{i}(t)$. The total number of Infectious individuals in city $i$ on day $t$ is $\sum_{\tau=1}^{\tau=\tau_{2}} I_{i}(\tau,t)$. Therefore, the total number of newly Exposed persons in city $i$ at the start of day $t+1$ is

$E_{i}(0, t+1)=\lambda\frac{S_{i}(t)}{T_{i}(t)}\sum_{\tau=1}^{\tau_{2}} I_{i}(\tau,t)$. (8)

### *Transportation Operator*

Let $A_{i}(t)$ represent the number of individuals in city $i$ on day $t$ in any of the groups allowed to travel, and let $\sigma_{ij}$ be the average daily number of travelers from city $i$ to city $j$. The net change in $A_{i}(t)$ due to travel to and from city $i$ can be included in a transportation operator $\Omega$:

$\begin{matrix} \Omega\left[A_{i}(t)\right]=\sum_{j=1}^{n} \left[A_{j}(t)\frac{\sigma_{ji}}{T_{j}(t)}-A_{i}(t)\frac{\sigma_{ij}}{T_{i}(t)}\right] \\ \text{ }=\sum_{j=1}^{n} \left[A_{j}(t)\cdot pT_{ji}(t)-A_{i}(t)\cdot pT_{ij}(t)\right] \end{matrix}$ . (9)

The first term in the summation in Equation (9) refers to individuals traveling to city $i$ from city $j$. The second term in the summation refers to individuals traveling from city $i$ to city $j$. Note that when $j=i$, the terms in the summation cancel and the net number of travelers from city $i$ to itself is zero.

To prevent the early occurrence of new epidemics in cities due to small fractions of Exposed individuals moving through the transportation network, the definition of $\Omega$ is modified slightly for Exposed travelers:

$\Omega'\left[E_{i}(\tau,t)\right]=\left\{\begin{matrix} \Omega\left[E_{i}(\tau,t)\right]\text{ if }\Omega\left[E_{i}(\tau,t)\right]\cdot\sum_{\tau=0}^{\tau_{1}} f(\tau)\geq1 \\ 0\text{ if }\Omega\left[E_{i}(\tau,t)\right]\cdot\sum_{\tau=0}^{\tau_{1}} f(\tau)<1 \end{matrix}\right.$ (10)

This modification ensures that Exposed individuals are allowed to travel to an unexposed city *i* only if the approximate expected total of these individuals is at least one.

For later notational convenience, we also define

(11)

to capture the net change in the total population of city $i$due to travel on day $t$.

### *Seasonality of Disease Transmission*

Diseases such as influenza often show seasonality in their transmission rates. We assume that there are both seasonal and geographical variations in the infectious contact rate, $\lambda$, and that these variations are continuous. We therefore define a scaling factor, $sf(l_{i}, t)$, to capture the variation in $\lambda$ in city $i$, based on the latitude $l_{i}$ of the city and on the time $t$:

$sf(l_{i}, t)=sf_{max}-\left(\frac{sf_{max}-sf_{min}}{2}\right)\cdot\sin\left(\frac{\pi\Lambda_{i}}{2}\right)\cdot\left[1-\mathrm{sgn}\left(l_{i}\right)\cos\left(\frac{2\pi\left(t+t_{0}\right)}{365.25}\right)\right]\text{ }$ (12)

where $t_{0}$ is the day of the year corresponding to the start date of the epidemic. In Equation (12),

$\Lambda_{i}=\min\left(1,\max\left(0,\frac{\left|l_{i}\right|-l_{min}}{l_{max}-l_{min}}\right)\right)$ . (13)

Thus, $\Lambda_{i}$ scales $l_{i}$ so that the amplitude of the seasonal variation in $\lambda$ changes continuously with latitude, with $sf\left(l_{i},t\right)=sf_{max}$ for cities located in the tropics $\left(0\leq\left|l_{i}\right|\leq l_{min}\right)$, increasing seasonal variation for cities located farther north or south $\left(l_{min}\leq\left|l_{i}\right|\leq l_{max}\right)$, and maximum seasonal variation of $sf_{min}\leq sf\left(l_{i},t\right)\leq sf_{max}$ for cities located at extreme latitudes $\left(\text{ }\left|l_{i}\right|\leq l_{max}\right)$.

### *Deterministic State Equations*

Before interventions or stochasticity are included in the model, the full set of state equations is thus:

$NS_{i}(t+1)=NS_{i}(t)+\Omega\left[NS_{i}(t)\right]$, (14)

$E_{i}(0,t+1)=\frac{sf(l_{i},t)\cdot\lambda\cdot\left(S_{i}(t)+\Omega\left[S_{i}(t)\right]\right) }{T_{i}(t)-D_{i}(t)+\omega\left[T_{i}(t)-D_{i}(t)\right]}\sum_{\tau=0}^{\tau_{2}} I_{i}(\tau,t),$ (15)

$S_{i}(t+1)=S_{i}(t)+\Omega\left[S_{i}(t)\right]-E_{i}(0,t+1)$, (16)

$E_{i}(\tau+1,t+1)=\left[1-\gamma(\tau)\right]\left(E_{i}(\tau,t)+\Omega\left[E_{i}(\tau,t)\right]\right),\text{ for }\tau=\text{0,1,...,}\tau_{1}-1$ (17)

$I_{i}(\tau+1,t+1)=\left\{\begin{matrix} \gamma(\tau)\left(E_{i}(\tau,t)+\Omega\left[E_{i}(\tau,t)\right]\right)+\left[1-\delta(\tau)\right]I_{i}(\tau,t),\text{ for }\tau=\text{0,1,...,}\tau_{1} \\ \left[\text{1-}\delta\text{(}\tau\text{)}\right]I_{i}(\tau,t),\text{ for }\tau=\tau_{1}+1,\tau_{1}+2,...,\tau_{2}-1 \end{matrix}\right.$ (18)

$R_{i}(t+1)=R_{i}(t)+\Omega\left[R_{i}(t)\right]+\left(1-d\right)\cdot\sum_{\tau=0}^{\tau_{2}} \delta(\tau)I_{i}(\tau,t)$, (19)

$D_{i}(t+1)=D_{i}(t)+d\cdot\sum_{\tau=0}^{\tau_{2}} \delta(\tau)I_{i}(\tau,t)$ , (20)

$T_{i}(t+1)=NS_{i}(t+1)+S_{i}(t+1)+\sum_{\tau=0}^{\tau_{1}} E_{i}(\tau,t+1)+\sum_{\tau=0}^{\tau_{2}} I_{i}(\tau,t+1)+R_{i}(t+1)+D_{i}(t+1)$ (21)

$W_{i}(t+1)=\sum_{\tau=0}^{\tau_{1}} \gamma(\tau)\left(E_{i}(\tau,t)+\Omega\left[E_{i}(\tau,t)\right]\right)$ (22)

$B_{i}(t+1)=\beta\cdot W_{i}(t+1)$ . (23)

The boundary conditions used with this set of equations are given by Equation (15) above and by $I_{i}(0,t)=0$.

The initial conditions for the model are given by

$E_{i}(0, 0)=\left\{\begin{matrix} \eta\cdot T_{i}(0)\text{ for }i=i_{0} \\ 0\text{ for }i\neq i_{0} \end{matrix}\right.$ , (24)

$NS_{i}(0)=(1-\alpha)\cdot T_{i}\text{(0) }\forall i$, (25)

$S_{i}(0)=\alpha\cdot T_{i}(0)-E_{i}(0, 0)\text{ }\forall i$, (26)

$E_{i}(\tau, 0)=0\text{ }\forall i\text{ , }\tau=1, 2, \ldots, \tau_{1}$ , (27)

$I_{i}(\tau, 0)=0\text{ }\forall i\text{ , }\tau=0, 1, \ldots, \tau_{2}$, (28)

$R_{i}(0)=0\text{ }\forall i$, (29)

$D_{i}(0)=0\text{ }\forall i$, (30)

$W_{i}(0)=0\text{ }\forall i$, (31)

$B_{i}(0)=0\text{ }\forall i$, (32)

where $\eta$ is the initially exposed fraction of the population, $T_{i}(0)$ is the initial population of city $i$, $i_{0}$is the index of the city initially exposed to the virus, and $\alpha$ is the initially susceptible fraction of the population.

***Stochasticity***

We included two potential sources of stochasticity in the model: random contact between individuals, and random travel from city to city. Randomness was applied to each of these processes in a way that accounts for the underlying nature of the process involved. The user can select whether contact is deterministic or stochastic independently of the choice for travel.

*Random Contact*

We assume that random contacts between pairs of individuals are independent of each other and that the number of new contacts that occur between two times, $t$ and $t+\Delta t$, does not depend either on the number of previous contacts or on the time $t$. Under these assumptions, the number of random contacts between individuals follows a Poisson distribution. In the deterministic case, the total number of infectious contacts per day is given by Equation (15). In the stochastic case, this equation then becomes

$E_{i}(0,t+1)=\tilde{C}$, with $\tilde{C}\text{\textasciitilde}\mathrm{Poisson}\left(\frac{sf(l_{i},t)\cdot\lambda\cdot\left(S_{i}(t)+\Omega\left[S_{i}(t)\right]\right)}{T_{i}(t)-D_{i}(t)+\omega\left[T_{i}(t)-D_{i}(t)\right]}\sum_{\tau=0}^{\tau_{2}} I_{i}(\tau,t)\right)$. (33)

The mean number of infectious contacts in the case of random contact is therefore equal to the number of infectious contacts in the deterministic case.

*Random Travel*

Another natural source of randomness in the model is the daily number of travelers between cities. Travelers from one city may travel to any one of multiple destinations. In the deterministic travel case, the model uses the appropriate average daily number of travelers for each connection from one city to another. In the random travel case, the numbers of travelers to each destination from a given city should be drawn from a multinomial probability distribution. This choice is implemented in the model as a series of draws from binomial distributions. The distribution used for each destination is based on both the number of potential travelers remaining in the origination city after the numbers of travelers to previous destinations in the list have been chosen, and the conditional probability of choosing that destination, given that no previous destination has been chosen. Travel is calculated separately for each disease state group that is permitted to travel.

Thus, if $A_{i}(t)$ is the initial number of potential travelers in a disease state in city $i$ at time $t$, $RA_{ij}(t)$is the remaining number of potential travelers after travel to the first $j-1$ destinations has been calculated, and $nT_{ij}(t)$ is the number of travelers from city $i$ to city $j$, then

$RA_{ij}(t)=\left\{\begin{matrix} A_{i}(t)\text{ if }j=1 \\ A_{i}(t)-\sum_{k=1}^{j-1} nT_{ik}\text{(}t)\text{ if }j>1 \end{matrix}\right.$ , (34)

, (35)

and

$cpT_{ij}(t)=\left\{\begin{matrix} pT_{ij}(t)\text{ if }j=1\text{ } \\ \frac{pT_{ij}(t)}{1-\sum_{k=1}^{j-1} pT_{ik}(t)}\text{ if }j>1\text{ } \end{matrix}\right.\text{ }$. (36)

Here $pT_{ij}(t)$ is the unconditional probability of travel from city $i$ to city $j$, as in Equation (9), and $cpT_{ij}(t)$ is the conditional probability of travel from city $i$ to city $j$ given that travel has not occurred from city $i$ to any of the previous $j-1$ destination cities. As with random contact, the mean number of travelers in the case of random travel is the same as the deterministic number of travelers to each destination.

If random travel is used, the transportation operator from Equation (9) is replaced by the following:

$\Omega\left[A_{i}(t)\right]=A_{i}(t)+\sum_{j=1}^{n} \left[nT_{ji}(t)-nT_{ij}(t)\right]$ . (37)

***Interventions***

***Travel Restrictions***

Travel restrictions can be imposed in the model in one of two ways: globally or sequentially. Under the global option, travel is reduced by some fraction $f_{r}$ to and from every city worldwide, if the total number of infectious cases in the initially exposed city reaches a certain threshold. Under the sequential option, travel is reduced to and from each city individually, if the total number of infectious cases within that city reaches the threshold for imposing travel restrictions. If travel restrictions are imposed on city *i*, then $pT_{ij}(t)$ in Equation (9) is replaced by

$pT_{ij, restricted}(t)=pT_{ij}(t)\cdot(1-f_{r})$. (38)

The travel restriction option may be implemented either at the start of or during a simulation. Travel restrictions may also be removed during a simulation, returning travel probabilities to their unrestricted values.

***Vaccination***

Vaccination has been used extensively for controlling disease by increasing the immunity level in the population. We have constructed two vaccination strategies in the model, which can be administered either separately or in combination: one-time vaccination and daily vaccination. We assume that the effectiveness of the vaccine among Susceptibles is VE_S_. As discussed earlier, we keep this parameter at 100 %. The first strategy is to vaccinate some fraction $v=f_{v1}$ of the Susceptible group at one time point during the simulation. The second strategy is to vaccinate some fraction $v=f_{v2}$ of the Susceptible group every day of the simulation while the strategy is in effect. Those vaccinated become Nonsusceptible. Equations (14) and (16) for the numbers of Nonsusceptible and Susceptible individuals are then replaced by

$NS_{i}(t+1)=NS_{i}(t)+\Omega\left[NS_{i}(t)\right]+VE_{S}\cdot v\cdot\left(S_{i}(t)+\Omega\left[S_{i}(t)\right]\right)$ (39)

and

$S_{i}(t+1)=S_{i}(t)+\Omega\left[S_{i}(t)\right]-E_{i}(0,t)-VE_{S}\cdot v\cdot\left(S_{i}(t)+\Omega\left[S_{i}(t)\right]\right)$ . (40)

Each vaccination option may be implemented either at the start of or during a simulation. If the two strategies are combined in the same simulation, then the daily vaccination occurs every day except on the day of the one-time vaccination. A vaccination strategy may also be stopped during a simulation.
