# Supplementary material for: Controlling Pandemic Flu: The Value of International Air Travel Restrictions
Source: PLoS One. 2007 May 2;2(5):e401. doi: 10.1371/journal.pone.0000401 (PMC1855004; doi:10.1371/journal.pone.0000401)
Supplement: Table S1 — Parameters and Values for the Model that Do Not Vary over Time (0.09 MB DOC) [file pone.0000401.s002.doc]

**Table S1 – Parameters and Values for the Model that Do Not Vary over Time**

| No. | Parameter Name | Description | Type | Default Value |
| --- | --- | --- | --- | --- |
| 1 | nReplications | Total number of model runs | Integer | 100 |
| 2 | nCities | Total number of cities in the model | integer | 155 |
| 3 | initExposedCity | Index # of the city initially exposed to the disease | integer | 53 |
| 4 | initExposedNumber | Number of individuals initially exposed to the disease | integer | 100 |
| 5 | startYear | Year that time series starts | integer | 2000 |
| 6 | startMonth | Month that time series starts | integer | 6 ( July )  ( 0 – 11 ↔ Jan–Dec ) |
| 7 | startDay | Day that time series starts | integer | 1 |
| 8 | endTime | Replication end time | integer | 365 days |
| 9 | animationScaleFactor | Factor to scale the animation to screen | real | 0.6 |
| 10 | populationFileName | Population input file | String | "population_155.txt" |
| 11 | travelFileName | Average daily passenger input file | String | "travel_155.txt" |
| 12 | outputDaily | Controls whether results are output daily or quarterly to the results file | Boolean | true |
| 13 | resultsFileName | Output file name | String | "global_results.csv" |
| 14 | appendToFile | Controls whether output data is appended to or overwrites file | Boolean | true |
| 15 | alpha | Fraction of population that is initially susceptible | real | 1 |
| 16 | beta | Fraction of newly infectious persons reported to the health registry | real | 0.3 |
| 17 | R0 | Basic reproduction number of virus | real | 1.7 |
| 18 | tau1 | Maximum day of the Exposed period | integer | 1 |
| 19 | tau2 | Maximum day of the Infectious period | integer | 7 |
| 20 | deathRate | Fraction of infected persons who die | real | 0 |
| 21 | interveneSequentially | Controls whether interventions are sequential or simultaneous | Boolean | true |
| 22 | interventionThreshold | Threshold of newly Infectious at which to apply interventions | real | 1000 |
| 23 | restrictTravel | Controls whether travel intervention is applied | Boolean | true |
| 24 | restrictionLevel | Fraction by which to restrict travel | real | 0.90 |
| 25 | quarantine | Controls whether quarantine is applied | Boolean | false |
| 26 | quarantineFraction | Fraction of Infectious who may be quarantined before recovery | real | 0.5 |
| 27 | vaccinateOneTime | Controls whether one-time vaccination occurs | Boolean | true |
| 28 | vaccinationFractionOneTime | Fraction of susceptibles to be vaccinated when one-time vaccination occurs | real | 0.1 |
| 29 | vaccinateDaily | Controls whether daily vaccination occurs | Boolean | false |
| 30 | vaccinationFractionDaily | Fraction of Susceptibles to be vaccinated daily | real | 0.001 |
| 31 | randomTravel | Controls whether travel is random | Boolean | true |
| 32 | randomContact | Controls whether number of infectious SI contacts is random | Boolean | true |
| 33 | useCernRNG | Controls whether CERN package or built-in AnyLogic™ code is used for Poisson and Binomial distributions | Boolean | true |
| 34 | minSF | Minimum value of seasonal scale factor | real | 0.1 |
| 35 | maxSF | Maximum value of seasonal scale factor | real | 1.0 |
| 36 | minLatN | Northern boundary of equatorial zone | real | 23.5 ° |
| 37 | maxLatN | Northern boundary of increasing northern seasonality | real | 66.5 ° |
| 38 | minLatS | Southern boundary of equatorial zone | real | - 23.5 ° |
| 39 | maxLatS | Southern boundary of increasing southern seasonality | real | - 66.5 ° |
