# Supplementary material for: Controlling Pandemic Flu: The Value of International Air Travel Restrictions
Source: PLoS One. 2007 May 2;2(5):e401. doi: 10.1371/journal.pone.0000401 (PMC1855004; doi:10.1371/journal.pone.0000401)
Supplement: Text S2 — Modifications to the Travel Matrix to Account for Multiple Legs of Travel (0.16 MB DOC) [file pone.0000401.s003.doc]

**Text S2 – Modifications to the Travel Matrix to Account for Multiple Legs of Travel**

Let $n$ cities be indexed by $i$, so that $i=1, 2, \ldots, n$.

Let $\mathbf{A}$ be the $n\times n$matrix with $ij^{th}$entry, $A_{ij}$, equal to the probability that a passenger from city $i$ travels to city $j$, such that $\sum_{j} A_{ij}=1$ (a passenger has to travel somewhere), and $A_{ii}=0$ (a passenger does not travel from a city to itself). Then $\mathbf{A}$ is the one-leg travel probability matrix. Note that the probability of travel from city $i$ to city $j$ is not necessarily equal to the probability of travel from city $j$ to city $i$, and so $\mathbf{A}$ is not necessarily symmetric.

If there are $x_{i}$ total passengers leaving each city $i$, then the number of travelers, $y_{j}$, arriving in each city $j$ after one leg of travel is $y_{j}=\sum_{i} \left(x_{i}A_{ij}\right)$.

To properly account for a passenger who is taking a two-leg trip from city $i$ through city $k$ to city $j$, we must ensure that that person does not return from city $k$ to city $i$ on the second leg of the trip, that is, that $j\neq i$. Let $B_{kj}$ be the probability of travel from city $k$ to city $j$ given that $j\neq i$. Then $B_{ki}=0$, and $B_{kj}=A_{kj}/\left(1-A_{ki}\right)$ for $j=1, \ldots, n$, $j\neq i$. Thus we have that the total number of travelers arriving in city $j$after two legs of travel is given by $z_{j}=\sum_{i, k} \left(x_{i}A_{ik}B_{kj}\right)=\sum_{i} x_{i}\left(\sum_{k} \left(A_{ik}B_{kj}\right)\right)$. We define $\mathbf{B}$ as the $n\times n$ matrix of two-leg travel probabilities, with the $ij^{th}$element equal to $\sum_{k} \left(A_{ik}B_{kj}\right)$.

We account similarly for travelers who arrive in a given city after a three-leg journey. A passenger traveling from city $i$ through cities $l$ and $k$ to arrive in city $j$ cannot return to either city $i$ or city $l$ from city $k$. Let $C_{kj}$ be the probability of travel from city $k$ to city $j$ given that $j\neq i, l$. Then $C_{ki}=C_{kl}=0$, and $C_{kj}=A_{kj}/\left(1-A_{ki}-A_{kl}\right)$ for $j=1, \ldots, n$, $j\neq i, l$. The total number of passengers arriving in city $j$ after three legs of travel is given by $w_{j}=\sum_{i, l, k} \left(x_{i}A_{il}B_{lk}C_{kj}\right)=\sum_{i} \left(x_{i}\left(\sum_{l, k} A_{il}B_{lk}C_{kj}\right)\right)$. We define $\mathbf{C}$as the $n\times n$ matrix of three-leg travel probabilities, with the $ij^{th}$element equal to $\sum_{l, k} \left(A_{il}B_{lk}C_{kj}\right)$.

Now let $p_{1, i}$ be the probability that a person from city $i$ travels exactly one leg to reach his destination, let $p_{2, i}$ be the probability that he travels two legs, and let $p_{3, i}$ be the probability that he travels three or more legs. Then the total probability of travel from city $i$ to city $j$ will be given by $D_{ij}=p_{1, i}\cdot A_{ij}+p_{2, i}\cdot A_{ik}B_{kj}+p_{3, i}\cdot A_{il}B_{lk}C_{kj}$. We define $\mathbf{D}$ as the $n\times n$ matrix with the $ij^{th}$element equal to $D_{ij}$. This matrix $\mathbf{D}$ is used as the multileg travel matrix in our global epidemic model.

We calculated the elements of $\mathbf{D}$ as follows:

Using a sample of U.S. travel itineraries, we fit the following model for the single-leg travel probabilities from each city $i$: $\text{logit}\left(p_{1, i}\right)=\beta_{0}+\beta_{1}\log\left(Pop_{i}\right)+\beta_{2}\log\left(Seats_{i}\right)$, where $Pop_{i}$ is the population of city $i$, and $Seats_{i}$ is the number of available outbound seats on flights from city $i$. We used this model to predict the probabilities of a single-leg trip for airports outside the United States, and collapsed the resulting distributions into three categories of airports, corresponding to small, medium, and large travel hubs. The three corresponding probabilities are $p_{1, i}=0.4$ for small hubs, $p_{1, i}=0.7$ for medium hubs, and $p_{1, i}=0.8$ for large hubs. Because the proportion of U.S. passengers traveling three or more legs to reach their destinations is less than 0.03, we set $p_{3, i}=0.02$. Finally, we set $p_{2, i}=1-p_{1, i}-p_{3, i}$, and calculated the elements of the matrix $\mathbf{D}$ as described above.

**Figure S1**. **Screenshot of the Global Epidemic Model Interface.** A user can select one of three visualization screens: a world map view, time series plots, or numeric tables for each of the cities. Before running the model, one can choose to produce stochastic or deterministic runs and choose the types of intervention. Each spot on the map corresponds to a metropolitan area. Clicking on a spot will display the city name and a snapshot of the city disease status. Arrows link each infected city with its initial source of infection.
