# Supplementary figures and images for: Controlling Pandemic Flu: The Value of International Air Travel Restrictions
Source: PLoS One. 2007 May 2;2(5):e401. doi: 10.1371/journal.pone.0000401 (PMC1855004; doi:10.1371/journal.pone.0000401)

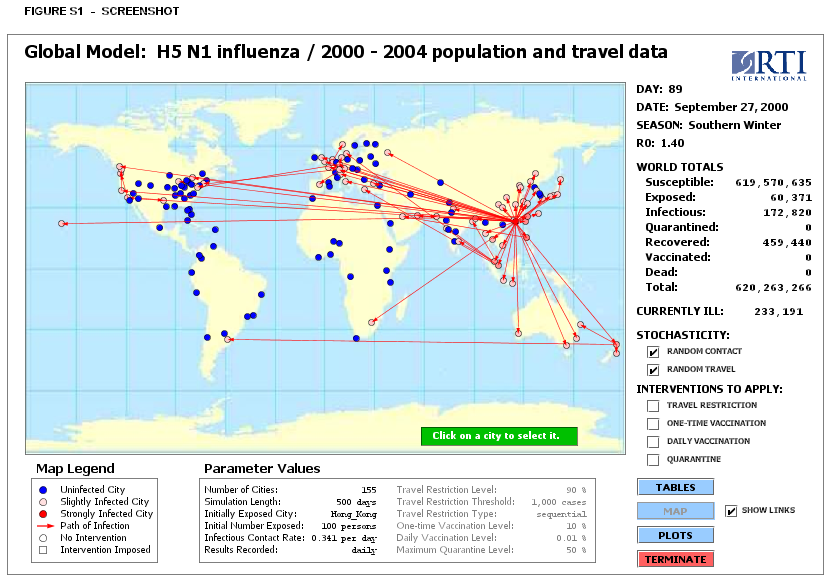

Supplement: Figure S1 — A user can select one of three visualization screens: a world map view, time series plots, or numeric tables for each of the cities. Before running the model, one can choose to produce stochastic or deterministic runs and choose the types of intervention. Each spot on the map corresponds to a metropolitan area. Clicking on a spot will display the city name and a snapshot of the city disease status. Arrows link each infected city with its initial source of infection. (0.45 MB TIF) [file pone.0000401.s004.tif]
